# The socioeconomic determinants of cancer

**DOI:** 10.1186/1476-069X-10-S1-S7

**Published:** 2011-04-05

**Authors:** Franco Merletti, Claudia Galassi, Teresa Spadea

**Affiliations:** 1 Center for Cancer Prevention, University of Turin, San Giovanni Battista University Hospital, Italy; 2 Regional Epidemiology Unit, ASL TO3 Piedmont Region, Grugliasco, Italy

## Abstract

This paper provides a synthesis on socioeconomic inequalities in cancer incidence, mortality and survival across countries and within countries, with particular focus on the Italian context; the paper also describes the underlying mechanisms documented for cancer incidence, and reports some remarks on policies to tackle inequalities.

From a worldwide perspective, the burden of cancer appears to be particularly increasing in developing countries, where many cancers with a poor prognosis (liver, stomach and oesophagus) are much more common than in richer countries. As in the case of incidence and mortality, also in cancer survival we observe a great variability across countries. Different studies have suggested a possible impact of health care on the social gradients in cancer survival, even in countries with a National Health System providing equitable access to care.

In developed countries, there is increasing awareness of social inequalities as an important public health issue; as a consequence, there is a variety of strategies and policies being implemented throughout Europe. However, recent reviews emphasize that present knowledge on effectiveness of policies and interventions on health inequalities is not sufficient to offer a robust and evidence-based guide to the choice and design of interventions, and that more evaluation studies are needed.

The large disparities in health that we can measure within and between countries represent a challenge to the world; social health inequalities are avoidable, and their reduction therefore represents an achievable goal and an ethical imperative.

## Introduction

“ When one reviews the literature, it is rather depressing to encounter the same observations, the same results, and the same conclusions and recommendations repeated over the years. Although there is not much to be found that is new, poverty continues to be rediscovered.” (Tomatis, 1992)

Even in developed countries, social factors account for a significant part of the total burden of disease in the population [1]. In the definition given by Krieger, “social inequalities… in health refer to health disparities, within and between countries, that are judged to be unfair, unjust, avoidable, and unnecessary… and that systematically burden populations rendered vulnerable by underlying social structures and political, economic, and legal institutions” [2].

In this paper we will briefly summarize the contents of the presentation on socio-economic determinants of cancer, given at the 1st Lorenzo Tomatis Conference on Environment and Cancer held in Turin (Italy) on June 4-5, 2009. The issue of socio inequalities in cancer was frequently faced by Lorenzo Tomatis [3,4], and by IARC researchers [5,6].

## Inequalities in cancer mortality and incidence

Of the estimated 12.7 million new cancer cases and 7.6 million cancer deaths in 2008, 56% of the former and 63% of the latter occur in the less developed countries. Among women cervical cancer is the third most common cancer overall, accounting for 13% of all female cancers; however, 85% of these new cases occur in developing countries [7].

A substantial volume of literature has been published after the first IARC comprehensive publication on social inequalities and cancer [6], showing that large social inequalities in cancer incidence and mortality do exist also within Western countries. The monograph reported significantly higher risks among socially disadvantaged people for cancers of the lung, stomach, upper aero-digestive tract (UADT), and cervical cancer; the relationship with social status appeared direct for colon cancer, bone neoplasm, melanoma, and malignant breast and ovarian cancer, while for all other cancers studied, the available evidence was inconsistent [8].

Analysing the relative impact of each cause of death on the excess of mortality observed among the less educated groups of population, a more recent comparative European study found that the relative contribution of cancer mortality was about 24% on the whole, ranging from less than 20% in Nordic countries to more than 30% in Mediterranean countries [9]. Other overviews also highlighted different patterns of inequalities in cancer mortality across European countries: hence, lung cancer showed the highest relative index of inequalities among Eastern and Continental European countries [10], while alcohol related and stomach cancers accounted for most of the social inequalities in cancer mortality among Southern European men [11,12].

The opportunity of explaining the origin of social inequalities in cancer and understanding the individual role of the different socioeconomic determinants that are involved in this process could lead to specific actions to tackle social inequalities and reduce their impact on health. In this respect, many studies have tried to disentangle the relative contribution of social circumstances across different stages of life: adult socioeconomic position, for example, suggests behavioural risk factors, such as smoking or alcohol consumption; while childhood social indicators hint at long-lasting exposures, such as higher probability of viral infections. Also, different socioeconomic indicators may indicate different mechanisms for social inequalities: occupation is related both to occupational exposures and material living standards, while education captures a person’s skills in preventing health damages and/or facing illness through more appropriate pathways of care [13].

Evidence from studies conducted in the last decade suggests that lung cancer risk is primarily associated either with adult socioeconomic position [14-16], or with measures of disadvantage during the whole life course [17-20]. This is generally attributed to inequalities in smoking and in occupational exposures: two studies found in fact that adjustment for smoking decreased educational differences in lung cancer incidence by 50 to 65% [21], while occupational exposures accounted for a residual 14% of inequalities after adjustment for smoking and diet [22]. The cultural (education) and material dimensions (occupation, housing, deprivation index) of disadvantage are influential, respectively, in the uptake and persistence of smoking [23]. Following the smoking epidemic model, strong age, gender and geographical differences in the social distribution of smoking have been observed [24]. Data indicate that an increase in smoking habit is still occurring among lower educated women in southern Europe, which, if not prevented, might result in even larger inequalities in smoking related cancers in the future.

Contrary to lung cancer, stomach cancer has been always associated with social circumstances during infancy and adolescence [14,17-20], consistently with the viral origin of this cancer. As for the other sites, most of them appear to be correlated with adulthood social characteristics [18,20], except for breast, cervical, and colorectal cancer that are also associated with early social circumstances [16,17,19].

Most of these studies, however, used cancer mortality as the outcome, although mortality also depends on access to health care for timely diagnosis and appropriate treatment, a confounding factor in the association between cancer and socioeconomic position.

In Italy, data from the linkage between the Turin Longitudinal Study and the Piedmont Cancer Registry offer the opportunity of giving more insight into the relationship between cancer incidence and socioeconomic position. A first study [25] estimated that 17% of incident cases among men in the lowest educational group were attributable to education, while the least educated women showed an 11% protection. Low education was associated with higher risks of UADT, stomach, lung, liver, rectal, bladder, central nervous system and ill-defined cancers in men, and with stomach, liver and cervical cancers in women. Vice-versa, less educated men had lower risks of melanoma, kidney and prostate cancers, and less educated women were less likely to be diagnosed with melanoma, ovarian and breast cancers. The only significant temporal changes in the educational gradients were observed among men, with an increase in rectal and bladder cancers and a decrease in kidney cancer risk for the most disadvantaged groups; also, men in the lowest group lost their initial protection for colon cancer.

A second study on the same data [26] compared the overall gradient associated to different indicators of socioeconomic economic position during early and late adulthood, by means of relative indexes of inequality (RIIs) (Table 1); this allowed assessing the independent effect of each indicator on cancer risk. Apart from confirming previous results from European studies, as for lung, stomach, breast and cervical cancers, this study highlighted some new and interesting results. First, it appeared that among men all the indicators of individual socioeconomic position have an equally strong independent impact on the overall risk of cancer, while the area-based deprivation index adds a smaller contribution. Second, among women the overall risk of cancer was significantly associated with education and housing characteristics in the two opposite directions: less educated women were protected, as their total risk mainly reflected the pattern of breast and lung cancer risk; the material dimension of disadvantage in adulthood, instead, was correlated to a higher risk of cervical cancer, and, unlike previous results, of lung cancer. Material indicators might in fact be those that better capture the reversing social gradient in smoking among women [27], because smoking could represent a way of facing economic difficulties and related stress [28]. Finally, male liver cancer appeared more associated with the material dimension of the socioeconomic position, possibly because of a health selection process by which people with alcohol dependence might be pushed downwards in the occupational hierarchy [29].

**Table 1:** Cancer incidence according to different socioeconomic indicators and selected cancer sites. Relative Index of Inequality (RII) with their 95% CI, estimated from the fully adjusted model^a^. Turin, 1985-99.

		ALL SITES		LUNG		STOMACH		UADT	LIVER	BREAST	CERVIX
		Men	Women	Men	Women	Men	Women	Men	Men	Women	Women
		n=33365	n=29834	n=7219	N=1656	n=1893	n=1212	n=2760	n=1726	n=9203	n=871

**Education** **RII (95% C.I.)**		**1.17** **(1.09-1.27)**	**0.78** **(0.72-0.85)**	**1.72** **(1.45-2.04)**	**0.54** **(0.37-0.77)**	**3.24** **(2.28-4.61)**	**2.16** **(1.34-3.48)**	**1.82** **(1.39-2.36)**	1.15 (0.81-1.63)	**0.63** **(0.55-0.72)**	**1.80** **(1.13-2.88)**
**Occupational Class** **RII (95% C.I.)**		**1.10** **(1.02-1.18)**	0.97 (0.90-1.05)	1.10 (0.94-1.27)	1.07 (0.74-1.52)	1.00 (0.74-1.36)	0.90 (0.58-1.40)	**1.60** **(1.27-2.02)**	**1.59** **(1.17-2.17)**	**0.87** **(0.77-0.99)**	1.09 (0.70-1.68)
**Housing Characteristics** **RII (95% C.I.)**		**1.26** **(1.18-1.34)**	**1.12** **(1.04-1.19)**	**1.72** **(1.51-1.95)**	**1.45** **(1.06-1.98)**	**1.33** **(1.02-1.73)**	1.13 (0.77-1.65)	**1.92** **(1.57-2.35)**	**1.37** **(1.05-1.80)**	1.06 (0.95-1.18)	**2.13** **(1.48-3.09)**
**Deprivation Index** **RII (95% C.I.)**		**1.09** **(1.03-1.16)**	0.98 (0.91-1.04)	**1.24** **(1.09-1.41)**	1.08 (0.79-1.47)	1.03 (0.79-1.34)	1.26 (0.86-1.84)	**1.38** **(1.13-1.68)**	0.94 (0.72-1.22)	0.92 (0.82-1.02)	1.30 (0.89-1.89)

**^a^**adjusted for age, area of birth and all the other reported variables from Spadea et al. [26]

An inverse association between cancer incidence and social deprivation has been observed for some cancer sites, including malignant melanoma, female breast cancer and prostate cancer [8,30]. Reproductive risk factors (such as later age at first pregnancy, lower parity, lower rates of breastfeeding, and a higher use of hormone replacement therapy) and a higher use of screening programmes are the most plausible explanations for the higher incidence of breast cancer among the less deprived socioeconomic groups [31,32]. Intermittent exposure to UV rays (e.g. during holidays) [33] and earlier diagnosis [34] are probably related to the higher incidence of melanoma among more advantaged people, while the higher incidence of prostate cancer among the less deprived is likely due to their larger use of opportunistic screening with PSA [35].

Shack et al, using data from all the English cancer registries in the period 1998-2003, estimated that if all socioeconomic groups had incidence rates similar to the least deprived group the number of cases would decrease by about 37% for lung cancer and 28% for cervical cancer, while it would increase by 7% for breast cancer and by about 28% for melanoma [36]. It should be noted, however, that recent trends in some reproductive behaviours, suggest a future reversal of breast cancer risk to the disadvantage of less educated women in various countries [37-39].

## Inequalities in cancer survival

As in the case of incidence and mortality, also in cancer survival we observe a great variability across countries: for example, the 5-year relative survival for breast cancer goes from above 80% in Cuba and USA to 40% in Algeria [40]. Not negligible differences have been reported also within Europe, particularly for elderly people and for specific cancer sites [41].

In the cited IARC Scientific Publication on Social Inequalities and Cancer, data had shown poorer survival for more disadvantaged groups of patients [42]. Relative risks ranged between 1.0 and 1.5, being largest for cancers with better prognosis, such as breast, body of uterus, bladder and colon. A more recent review, including studies conducted from 1995 to 2004, substantially confirmed the same scenario [43].

Given the high consistency of results across different populations, cancer sites, time periods, and socioeconomic indicators, artifactual explanations that have been suggested in the past, such as possible confounding or lead-time bias [44], appear now less likely or at most less influential. More likely explanations can be classified into three broad groups of factors: cancer stage at diagnosis, characteristics of the patients, and features related to the health care process. They have been extensively discussed in the cited review [43], and we will limit our discussion here to the most prominent conclusions.

Early stage at diagnosis is by far the most studied factor: it is the most important prognostic factor for survival, particularly for cancers with better prognosis, and it has been widely recognised as differently distributed across socioeconomic classes. Generally, the observed differences have been attributed to greater delay in seeking care among patients of lower socioeconomic status, compared with better-off patients, which are usually more health conscious. Among others, also Turin data showed that diagnostic delay and more advanced stages are more common in less educated people for colorectal cancer [45,46]. However, not always differences in stage at diagnosis were sufficient to fully explain the observed social gradients in survival and other factors (such as those discussed below) deserve to be more thoroughly analysed.

The second set of explanations – patient’s characteristics – relies on the hypothesis that socially disadvantaged patients could be more susceptible to the aggressiveness of cancer, or less responsive to treatment. A few studies have explored this hypothesis, however, and it’s impossible to quantify the impact of these factors on the social differentials in survival. The higher comorbidity associated with low social status could also contribute in reducing cancer survival; results concerning the association between low social support and lower survival for different chronic conditions, including cancer, are quite more consistent [47,48].

Finally, many studies have highlighted the role of health care in modifying the probability of cancer survival. Even in developed countries, with a National Health System providing equitable access to care for all patients, there is increasing evidence that part of the social gradients in survival could be explained by different treatments offered to patients in different socioeconomic groups. In England, for example, women with breast cancer living in deprived areas were less likely to undergo surgery and to receive breast-conserving surgery, and they had significantly lower 5-year survival than women in more affluent areas, also after controlling for stage at diagnosis [49]. A similar study in Italy did not find a significant effect on the type of surgery of the educational level of patients, but it highlighted a strong association with other non clinical factors, such as hospital volume of breast surgery, distance of woman’s residence from the nearest radiotherapy facility and marital status [50]. Both educational level and marital status were also strong predictors of the treatment received by lung cancer patients in Turin [51], as well as area deprivation was associated with poorer treatment and lower survival in London [52].

## Disparities after cancer

Social inequalities can be generated, or increased, among people who survive after a diagnosis of cancer. The experience of cancer and its treatment, especially during childhood and adolescence, may have long term consequences that can be potentially disrupting for the educational attainment and social functioning of subjects [53], and this, in turn, may increase the risk of health disparities. In a study based on a mailed survey completed by parents [54], survivors of childhood cancer were more likely to require special education programs, to attend learning-disability services, to repeat a grade, and to have educational or other school problems when compared to healthy controls. Furthermore, more survivors than controls had no close friends and did not use friends as confident. Indeed, several studies have reported a negative impact of childhood cancer on the marital status or on sexual relationship [53,55]. In a systematic review and meta-analysis [56], the risk of being unemployed among adult survivors of childhood cancer was almost doubled compared to healthy controls, and higher risks were found among survivors of central nervous system and brain tumours. Furthermore, the risk of unemployment was higher among survivors of childhood cancer in the United States than in comparable populations in Europe; the possible explanation for this finding can be the differences in social security and health care systems between Europe and US. Similar results have been recently found in a meta-analysis among subjects survived to a cancer diagnosed during working life: cancer survivors were found to have a 40% increased risk of being unemployed compared to healthy controls [57].

The prevalence of cancer survivors is expected to increase in most countries due to several factors, mainly the increasing early diagnosis, the continued improvement in cancer therapies, and the ageing of the population [58,59]; this supports the need to develop and evaluate interventions aimed at improving educational and social outcomes of cancer survivors.

## Can we reduce health disparities?

Social, economical and political factors have been clearly identified at the root of much of inequalities in health. Both distal and proximal causes of inequalities are involved, pointing to both upstream and downstream policies, from national economic strategies to local health prevention programmes [1,60]. Interventions and policies to be taken in order to advance health equity have been recently recommended by the Commission on Social Determinants of Health set up by the World Health Organization (WHO 2008). Three principal actions have been identified by the Commission: 1) improve the conditions of daily life; 2) tackle the inequitable distribution of power, money and resources; 3) measure and understand the problem and assess the results of actions. These actions are obviously of concern to all policy makers, not merely those involved in health policies.

In developing countries, the absolute number of cancer cases is expected to increase in the next decades [7]. Apart from aging of the population, other relevant factors are related both to the adaptation of developing countries to a western lifestyle, with a likely new epidemic of risk factors (such as smoking and unhealthy dietary habits) among the more disadvantaged, and to an increased exposure to occupational and environmental risks. At this regard, for example, asbestos-related diseases are expected to increase in developing countries, since these have been targeted by the asbestos industry after the ban (or serious restriction) of the use of asbestos in most developed countries [61]. From this point of view, the Rotterdam Convention could represent a relevant tool to protect countries where regulations on the use of hazardous chemicals are weak [62].

In developed countries, there is increasing awareness of social inequalities as an important public health issue; as a consequence, there is a variety of strategies and policies being implemented throughout Europe [63]. Some recent reviews on the effectiveness of policies include also interventions not directly aimed at reducing inequalities, but likely to impact positively on equity [64,65]. These reports, however, emphasize that present knowledge on effectiveness of policies and interventions on health inequalities is not sufficient to offer a robust and evidence-based guide to the choice and design of interventions, and that more evaluation studies are needed.

Among cancer-related policies, for example, the existence of disparities in the use of cancer screening procedures has been demonstrated since long, also in countries in which a universal insurance coverage was present [66-68]. However, a recent review has shown that socio-economic position inequalities in screening are lower in countries with nationwide population-based screening programmes, especially when compared to countries in which only opportunistic screening was present [69]. The available evidence supports the capability of some strategies (in particular: offering free tests, eliminating geographical barriers, a greater involvement of primary care physicians and individually tailored communication) in enhancing access to screening among lower socioeconomic groups [70]. This supports the fact that preventive measures and interventions may need innovative and bespoke approaches to reach in a more effective way the most vulnerable subgroups and the most deprived strata of the population [65,71]. As another example, policies supporting family building (e.g. paid parental leave, childcare services) might positively affect parity, maternal age and breastfeeding rates [65], thus contributing to prevent breast cancer incidence among lower socioeconomic groups.

Finally, it should be remembered that immigration from developing countries will increase the issue of inequalities in the rich countries; in fact, migrants often share common disadvantages, such as poverty, social isolation and psychosocial risk factors, as well as difficulties of access to health prevention and healthcare services. Future research on reducing cancer inequalities should therefore also take into account health needs of migrating populations.

In conclusion, the large disparities in health that we can measure within and between countries represent a challenge to the world; social health inequalities are avoidable, and their reduction therefore represents an achievable goal and an ethical imperative [72].

## Competing interests

The authors declare that they have no competing financial or non-financial interests.
